# Treatment of Single Patient With PMM2‐Congenital Disorder of Glycosylation With Govorestat (AT‐007), an Aldose Reductase Inhibitor

**DOI:** 10.1002/jmd2.70043

**Published:** 2025-10-12

**Authors:** Elizabeth R. Jalazo, Leigh Anne Weisenfeld, Anna Ligezka, Riccardo Perfetti, Joseph Muenzer

**Affiliations:** ^1^ University of North Carolina at Chapel Hill, Department of Pediatrics Chapel Hill North Carolina USA; ^2^ Department of Genetic and Genomic Sciences Mayo Clinic Rochester Minnesota USA; ^3^ Applied Therapeutics New York New York USA

**Keywords:** aldose reductase inhibitors, govorestat, PMM2‐CDG

## Abstract

Aldose reductase inhibitors (ARI) have been identified as a potential treatment for phosphomannomutase‐2 congenital disorder of glycosylation (PMM2‐CDG), a serious condition for which no treatments are approved. We treated a single patient for 36 months 30 months of age at enrollment, under a single‐patient investigational new drug expanded access request, with govorestat (AT‐007), a novel, highly selective, once daily, brain penetrant ARI at a starting dose of 1 mg/kg oral suspension, which was escalated to 30 mg/kg. The primary endpoint was safety. Secondary assessments included liver transaminases, factor XI, antithrombin III, and whole blood and urine sorbitol. Clinical outcomes were also assessed, including Nijmegen Pediatric CDG Rating Scale (NPCRS), Bayley Scales of Infant Development, and Vineland Adaptive Behavioral Scale. Govorestat was well tolerated; no adverse effects were noted. Aspartate aminotransferase (AST) and alanine aminotransferase (ALT) levels improved from a pre‐treatment 12‐month average of 205 and 268 U/L to 63 and 68 U/L, respectively, averaged over 36 months of govorestat treatment at 30 mg/kg. Antithrombin III and factor XI fluctuated with illness throughout the study period but overall increased by 60%–100%, approaching the normal range (> 83% activity) at the 5 mg/kg dose. Whole blood sorbitol decreased in a dose‐dependent fashion, normalizing at the 30 mg/kg dose. The NPCRS improved by 9 points (46%) over the course of treatment. In conclusion, our patient tolerated govorestat without safety concerns. Improvements in liver transaminases, clotting factors, and whole blood sorbitol were observed along with improvements in clinical measures. These findings support further study of govorestat as a potential treatment for PMM2‐CDG.

**Summary**

- PMM2‐congenital disorder of glycosylation
- Aldose reductase inhibitor
- Expanded access use
- Novel therapeutics
- Neurological disorders

## 1 Introduction

Phosphomannomutase‐2 congenital disorder of glycosylation (PMM2‐CDG) is a rare autosomal recessive multisystem genetic disorder caused by loss of PMM2 enzyme activity due to biallelic variants in the *PMM2* gene [1, 2]. The overall prevalence of genetic diseases affecting protein glycosylation has been reported as of3000 to 30 000 persons in the US, with an estimated prevalence of 1/20 000 in Europeans, 1/288 000 in Africans, and 1/367 000 in Asians [1, 3]. Approximately 1000 patients worldwide are reported with PMM2‐CDG though many more are suspected with an expected frequency of up to 1:20000 [2]. PMM2‐CDG is characterized by a spectrum of clinical involvement from an infantile multisystem phase with cognitive impairment to a stable adult disease with disability. Clinical manifestations and disease severity vary, but individuals with PMM2‐CDG often have liver dysfunction worsened by illness and metabolic stressors, clotting derangements with risk of both bleeding and thrombosis, stroke‐like episodes of unclear etiology, ataxia limiting mobility, global developmental delay, and intellectual impairment [1, 2].

The PMM2 enzyme performs an essential role in the N‐linked glycosylation of proteins, a process that is evolutionarily conserved because some degree of glycosylation is required at all times in all cellular tissues throughout development until the end of life. PMM2 catalyzes the conversion of mannose‐6‐phosphate to mannose‐1‐phosphate [2].

Currently, no treatments have been approved for PMM2‐CDG. Aldose reductase inhibitors (ARIs) were identified in a novel worm model, drug‐repurposing screen to have potential therapeutic benefit in PMM2‐CDG [4, 5]. Aldose reductase inhibition may shunt glucose metabolism away from the polyol pathway to glucose‐1,6‐bisphosphate, which is an endogenous stabilizer of the phosphomannomutase‐2 enzyme [6]. Previously developed ARIs inhibited aldehyde reductase—an enzyme responsible for detoxification of biomolecules in the liver—as well as the structurally similar target, aldose reductase, and thus were not developed further due to their lack of target specificity [7, 8]. In contrast, govorestat, a novel, highly selective, brain‐penetrant ARI in late‐stage clinical development for classical galactosemia and sorbitol dehydrogenase (SORD) deficiency, has high specificity for aldose reductase, with no off‐target aldehyde reductase inhibitory activity. Govorestat has been shown to have a pharmacokinetics profile that supports a once daily dosing regimen [9]. In vitro studies have shown increased PMM2 enzyme activity following treatment with govorestat, confirming findings with previously developed ARIs [4, 5]. Based on these observations, we sought to explore the safety and tolerability of govorestat in a single patient with PMM2‐CDG. We also explored potential markers of clinical improvement.

## 2 Materials and Methods

Under a single‐patient investigational new drug (IND) expanded access request, we treated a single male patient, age 30 months at enrollment, with govorestat.

PMM2 enzyme activity was evaluated in fibroblasts derived from the study patient and cultured according to the method previously described [5].

Govorestat was administered as an oral suspension once daily after at least 8 h of fasting (typically first thing in the morning prior to breakfast). We began treatment with a starting dose of 1 mg/kg and escalated to a target dose of 30 mg/kg while closely monitoring safety parameters, including liver and clotting function.

The primary endpoint was safety. Secondary exploratory endpoints included biochemical testing, including factor XI, antithrombin III, aspartate aminotransferase (AST) and alanine aminotransferase (ALT); serum transferrin isoelectrofocusing; PMM enzyme activity in fibroblasts; and whole blood and urine sorbitol. Whole blood sorbitol was measured with a validated LC–MS/MS assay.

The calibration range of the method is 25.0–5000 ng/mL using a 50.0 μL sample aliquot. The level of sorbitol in whole blood samples from fasting healthy adults ranges between200 and 400 ng/mL.

Clinical outcome measures included growth as assessed by pediatric weight percentile, the Nijmegen Pediatric CDG Rating Scale (NPCRS), Bayley Scales of Infant and Toddler Development, 3rd and 4th Editions (BSID‐III, BSID‐4), and the Vineland Adaptive Behavior Scales, 3rd Edition (VABS‐3) [10, 11, 12, 13, 14, 15].

## 3 Results

Prior to the administration of the first dose of govorestat, PMM2 enzyme activity in fibroblasts cultured in the absence of govorestat was 211 nmol/h/mg, while in those exposed to govorestat, the value was 298 nmol/h/mg, corresponding to a ~40% increase in enzyme activity.

**FIGURE 1:**
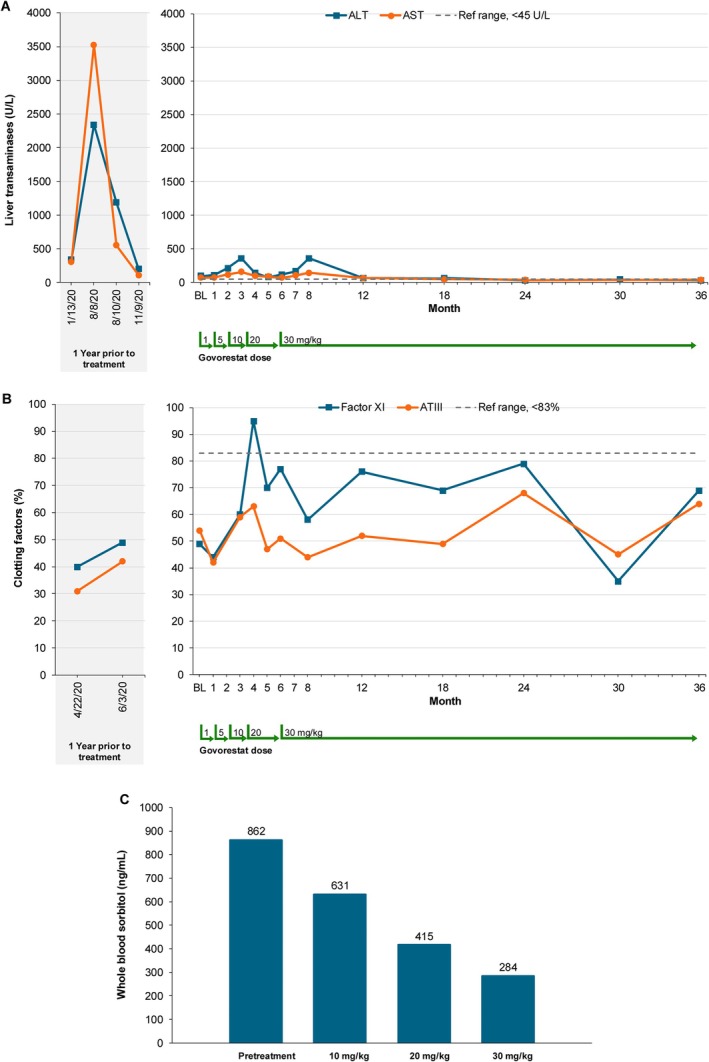
Changes in biochemical parameters during the 12 months prior to baseline and at subsequent timepoints during the study. Ref range = reference range. (A) Average level of aspartate aminotransferase (AST) and alanine aminotransferase (ALT). (B) Antithrombin III (AT‐III) and factor XI (FXI) as percentages of normal. (C) Dose‐dependent changes in whole blood sorbitol levels.

Over 36 months of treatment, govorestat was well tolerated with no notable safety concerns. There was no evidence of hepatotoxicity (Figure 1A). Over the 12 months prior to starting treatment, the participant's baseline AST and ALT averaged between 205 and 268 U/L when he was clinically well. Of note, he was hospitalized with confirmed HHV‐6 viral illness prior to initiation on govorestat. With this illness, he experienced a significant increase in serum transaminases, with AST/ALT > 3000 U/L. If considering these values, his 12‐month prior liver function was considerably variable with average AST and ALT 1122 and 1016 U/L respectively; at the remission of the viral illness, his AST/ALT average was at3–5 times the upper limit of normal before starting govorestat.

At target dose of 30 mg/kg govorestat, AST and ALT levels averaged 63 and 68 U/L, respectively. Variability in ALT and AST levels occurred with concurrent viral illnesses while on treatment with govorestat, but they never reached levels observed prior to the initiation of treatment with govorestat (Figure 1 Panel A). Typically, the AST and ALT levels decreased as the subject recovered from the viral illness. No adverse effects were noted, and govorestat was well tolerated by the subject. Early in the treatment period, prior to achieving target dose, the subject experienced an episode of lethargy and altered mental status, which prompted hospitalization for evaluation and observation. Encephalopathy resolved without intervention, and the subject was discharged following 24‐h observation with normal EEG and MRI findings redemonstrating known cerebellar hypoplasia. The subject had one additional emergency room visit during the study period for an unwitnessed fall with no associated loss of consciousness, but complaints of headache and a single episode of emesis. A Head CT was performed and was unremarkable, and he was discharged home without further intervention. Additional adverse events noted through the study period include upper respiratory infections, acute gastroenteritis, and febrile illnesses commensurate with what would be expected in a pediatric population during 36 months, none of which required hospitalization or intervention during the study period. No adverse events or serious adverse events were thought to be related to the study drug according to the investigator.

Antithrombin III and factor XI increased approaching the normal range at the 5 mg/kg dose (Figure 1B). Dose‐dependent reductions in whole blood sorbitol were observed, with normalization at 30 mg/kg (Figure 1C). Urine sorbitol levels were within the normal range prior to the initiation of treatment and were further reduced to 8 mmol/creatinine while on treatment. The blood sample collection for measurement of sorbitol was performed in the fasting state to limit any potential interference that could derive from the diet. No previous food‐effect studies have been conducted to support the administration of AT007 in the fed state, as such dosing was recommended in the fasted state. No changes in the patient's diet were made during the study, other than ensuring that morning dosing was performed following a period of at least 8 h of fasting overnight, just after waking and ~30 min before eating breakfast.

**FIGURE 2:**
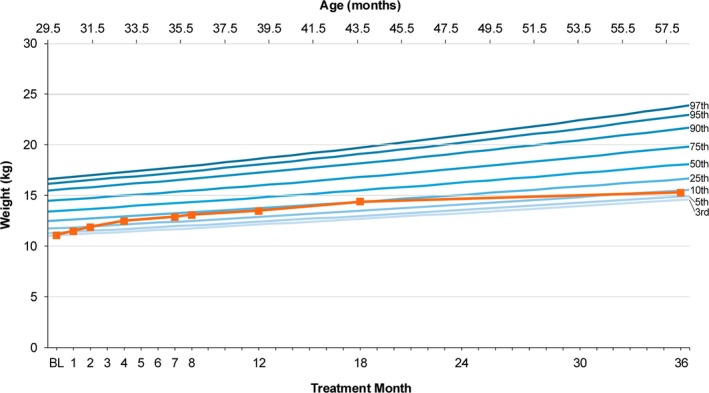
Growth (in kg) of study participant over the treatment period.

Prior to treatment, the participant exhibited poor growth velocity with a weight consistently below the 3rd percentile in 12 months prior to the initiation of govorestat. Weight increased during treatment and increased to the 10th percentile during the 36 months of treatment (Figure 2).

**FIGURE 3:**
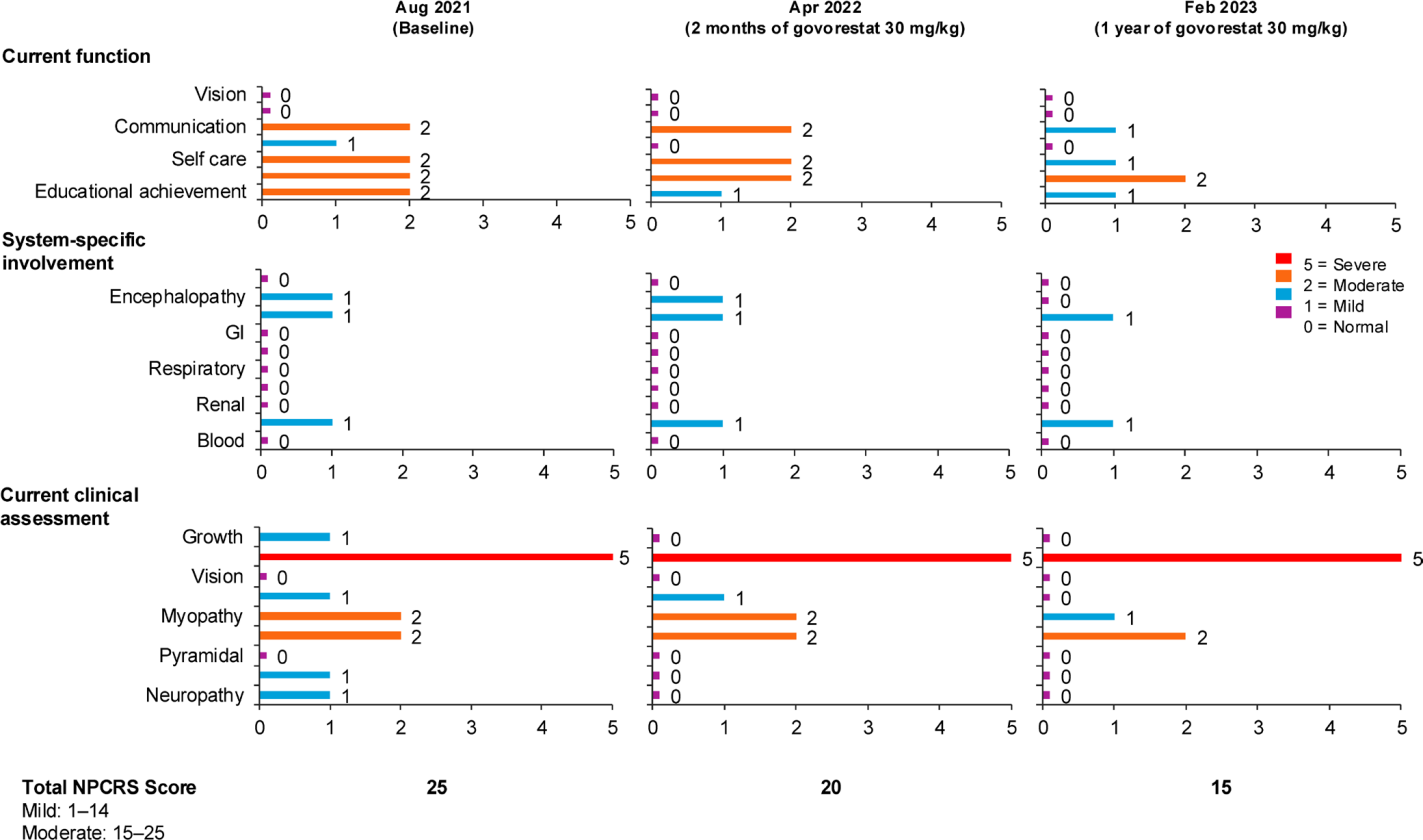
Results from the Nijmegen Progression CDG Rating Scale (NPCRS) assessment of study participants over the treatment period.

The NPCRS improved by 9 points (46%), from a baseline of 25–15 over the course of 36 months on govorestat (Figure 3).

The subject was initially evaluated for his baseline visit at the age of 29 months (2 years, 5 months), with follow‐up occurring when he reached the target drug dose at 37 months (3 years, 1 month) and then again at 69 months (5 years, 9 months). Both BSID and VABS‐3 indicated that some developmental improvement occurred, although the patient continued to function at a level below what would be expected at his given age (Table 1). While we are encouraged that our subject continued to make developmental strides during this study, we note that natural history studies of PMM2 deficiency suggest that developmental gains are expected over this period of observation and a direct relationship between treatment and clinical improvement cannot be established based on this open label single patient study.

**TABLE 1:** Results from Bayley scale of infant and toddler development and the vineland adaptive behavioral scales.

Bayley scales of infant and toddler development			
BSID subtest	Age equivalent at 29‐month evaluation^a^	Age equivalent at 37‐month evaluation^b^	Age equivalent at 69‐month evaluation^b^
Cognitive	17 months	25 months	34 months
Receptive language	16 months	28 months	30 months
Expressive language	13 months	21 months	27 months
Fine motor	16 months	17 months	31 months
Gross motor	9 months	10 months	12 months

Vineland adaptive behavior scales			
VABS‐3 subdomain	Age equivalent at 29‐month evaluation	Age equivalent at 37‐month evaluation	Age equivalent at 69‐month evaluation
Receptive language	13 months	26 months^c^	24 months^c^
Expressive language	9 months	17 months	23 months
Written	n/a	< 36 months	38 months
Personal	10 months	15 months	21 months
Domestic	n/a	< 36 months	< 36 months
Community	n/a	< 36 months	< 36 months
Interpersonal relationships	10 months	13 months	16 months
Play and leisure	10 months	16 months	21 months
Coping skills	< 25 months	< 24 months	< 24 months
Fine motor	16 months	22 months	37 months
Gross motor	12 months	12 months	12 months

^a^ BSID‐III.

^b^ BSID‐4.

^c^ Although subject age equivalent dropped slightly between these two timepoints, the discrepancy is minor.

## 4 Discussion

PMM2‐CDG has serious, life‐long impacts on growth and development, with no currently approved therapy. In most PMM2‐CDG cases, neurologic symptoms manifest soon after birth, including strabismus and abnormal eye movements, cerebellar hypoplasia, hypotonia, ataxia, and hyporeflexia. Up to 20% of infants die from multiorgan failure associated with serious liver, kidney, and cardiac conditions within the first year of life. In addition to phenotypic features such as strabismus, inverted nipples, and abnormal fat pads, affected children may develop retinitis pigmentosa, stroke‐like episodes, and seizures, speech and motor delays, and peripheral neuropathy. Although some of these unique features, like abnormal fat pad deposition in children, may resolve with age, adults continue to have neurological involvement, including cognitive impairment, peripheral neuropathy, and cerebellar ataxia, as well as musculoskeletal and endocrine abnormalities. In addition, decreased serum coagulation factors may put patients at increased risk of thrombosis and easy bleeding [2].

To date, govorestat has been safely used in clinical trials involving patients with classic galactosemia and SORD deficiency. The child with PMM2‐CDG treated in this study has tolerated govorestat for more than 36 months without significant safety signals. We also observed improvements in biochemical parameters, including liver transaminases, clotting factors, and whole blood sorbitol. Clinical outcome assessments further suggest early signs of improvement in the NPCRS clinical severity scale with a final rating (15) nearing the mild disease severity category (0–14).

Although our single patient on govorestat continues to function at a level below what would be expected at his given age, the age equivalents from both the BSID and VABS continue to move in a positive direction over time. Outcomes from his three evaluation timepoints indicate that he continues to gain new skills as he develops. Several areas of the patient's development show particularly notable skill acquisition. According to both his BSID and VABS outcomes, the patient's ability to both understand (receptive language) and communicate (expressive language) increased during the course of the study, indicating a trajectory of continued learning. Additionally, the patient gained many new fine motor skills during the course of the study time period. Coupled with his language growth, the patient's new skills have enabled him to function more independently in both the home and school environments.

A previous study with epalrestat, an older‐generation ARI, was conducted in a child with PMM2‐CDG. After 12 months, epalrestat treatment improved PMM2 enzyme activity, glycosylation biomarkers, NPCRS score, ataxia, and growth measures [4]. Epalrestat is approved in some Asian countries for the treatment of diabetic neuropathy, but it has not been approved for any indication in the United States or Europe [7, 16]. Epalrestat requires multiple daily administrations due to its short half‐life, while govorestat is administered once daily. Govorestat has high specificity for aldose reductase and therefore may be less likely to cause liver dysfunction. Indeed, in our study, AST and ALT levels improved during govorestat treatment. While spontaneous improvement of transaminases has been documented in PMM2‐CDG [17, 18], the observation from this trial is particularly relevant as first‐generation ARIs were often associated with liver toxicity.

This was an exploratory study in a single individual and therefore conclusions are limited. Nevertheless, our observations that govorestat was well tolerated and appeared to improve clinical manifestations of PMM2‐CDG warrant further study of this compound in PMM2‐CDG.

## Author Contributions

Elizabeth R. Jalazo and Riccardo Perfetti designed the study. Leigh Anne Weisenfeld conducted some of the clinical assessments. Joseph Muenzer oversaw the study conduct. All authors contributed to the writing of the manuscript.

## Disclosure

Govorestat is safe and well tolerated in a pediatric patient with PMM2‐CDG who experienced improvement of signs and symptoms during the 36 months duration of the study.

## Ethics Statement

All procedures followed were in accordance with the ethical standards of the responsible committee on human experimentation (institutional and national) and with the Helsinki Declaration of 1975, as revised in 2000.

## Consent

Written consent was obtained from the parents of the patient enrolled in the study.

## Conflicts of Interest

Anna Ligezka, Elizabeth R. Jalazo, Leigh Anne Weisenfeld, and Joseph Muenzer have no conflicts of interest. Riccardo Perfetti is an employee of Applied Therapeutics.

## Data Availability

The data that support the findings of this study are available from the corresponding author upon reasonable request.
